# AI-Powered Diagnosis of Skin Cancer: A Contemporary Review, Open Challenges and Future Research Directions

**DOI:** 10.3390/cancers15041183

**Published:** 2023-02-13

**Authors:** Navneet Melarkode, Kathiravan Srinivasan, Saeed Mian Qaisar, Pawel Plawiak

**Affiliations:** 1 School of Computer Science and Engineering, Vellore Institute of Technology, Vellore 632014, India; 2 Electrical and Computer Engineering Department, Effat University, Jeddah 22332, Saudi Arabia; 3 LINEACT CESI, 69100 Lyon, France; 4 Department of Computer Science, Faculty of Computer Science and Telecommunications, Cracow University of Technology, Warszawska 24, 31-155 Krakow, Poland; 5 Institute of Theoretical and Applied Informatics, Polish Academy of Sciences, Bałtycka 5, 44-100 Gliwice, Poland

**Keywords:** artificial intelligence, computer-aided diagnostics, deep learning, dermatologists, dermatology, digital dermatology, machine learning, man-machine systems, skin cancer, skin neoplasms

## Abstract

**Simple Summary:**

The proposed research aims to provide a deep insight into the deep learning and machine learning techniques used for diagnosing skin cancer. While maintaining a healthy balance between both Machine Learning as well as Deep Learning, the study also discusses open challenges and future directions in this field. The research includes a comparison on widely used datasets and prevalent review papers discussing skin cancer diagnosis using Artificial Intelligence. The authors of this study aim to set this review as a benchmark for further studies in the field of skin cancer diagnosis by also including limitations and benefits of historical approaches.

**Abstract:**

Skin cancer continues to remain one of the major healthcare issues across the globe. If diagnosed early, skin cancer can be treated successfully. While early diagnosis is paramount for an effective cure for cancer, the current process requires the involvement of skin cancer specialists, which makes it an expensive procedure and not easily available and affordable in developing countries. This dearth of skin cancer specialists has given rise to the need to develop automated diagnosis systems. In this context, Artificial Intelligence (AI)-based methods have been proposed. These systems can assist in the early detection of skin cancer and can consequently lower its morbidity, and, in turn, alleviate the mortality rate associated with it. Machine learning and deep learning are branches of AI that deal with statistical modeling and inference, which progressively learn from data fed into them to predict desired objectives and characteristics. This survey focuses on Machine Learning and Deep Learning techniques deployed in the field of skin cancer diagnosis, while maintaining a balance between both techniques. A comparison is made to widely used datasets and prevalent review papers, discussing automated skin cancer diagnosis. The study also discusses the insights and lessons yielded by the prior works. The survey culminates with future direction and scope, which will subsequently help in addressing the challenges faced within automated skin cancer diagnosis.

## 1. Introduction

Skin cancer is the abnormal growth of skin cells. The cancerous growth may affect both the layers—dermis and epidermis, but this review is concerned primarily with epidermal skin cancer; the two types of skin cancers that can arise from the epidermis are carcinomas and melanomas, depending on their cell type—keratinocytes or melanocytes, respectively [1,2,3,4,5,6,7,8,9,10,11,12,13,14,15,16,17,18,19,20,21,22,23,24,25,26,27,28,29,30,31,32,33,34,35,36,37,38,39,40,41,42,43,44,45,46,47,48,49,50,51,52,53,54,55,56,57,58,59,60,61,62,63,64,65,66,67,68,69,70,71,72,73,74,75]. It is a challenge to estimate the incidence of skin cancer due to various reasons, such as the multiple sub-types of skin cancer [76,77,78,79,80,81,82,83,84,85,86,87,88,89,90,91,92,93,94,95,96,97,98,99]. This poses as a problem while collating data, as non-melanoma is often not tracked by registries or are left incomplete because most cases are treated via surgery. As of 2020, the World Cancer Research Fund International reported a total of 300,000 cases of melanoma in skin, and a total of 1,198,073 cases of non-melanoma skin cancer [100,101,102,103,104,105,106,107,108,109,110,111,112,113,114,115,116,117,118,119,120,121,122,123,124,125,126,127,128,129,130,131]. The reasons for the occurrence of skin cancer cannot be singled out, but they include and are not limited to exposure to ultraviolet rays, family history, or a poor immune system [126]. The affected spot on the skin is called a lesion, which can be further segregated into multiple categories depending on its origin [1]. A comparison between different lesion types is usually accompanied by the presence or the absence of certain dermoscopic features. 

There are three stages associated with an automated dermoscopy image analysis system, namely pre-processing, image segmentation, and feature extraction [2,4]. Segmentation plays a vital role, as the succeeding steps are dependent on this stage’s output. Segmentation can be carried out in a supervised manner by considering parameters such as shapes, sizes, and colors, coupled with skin texture and type. Melanoma development that takes place horizontally or radially along the epidermis is called “single cancer melanoma”, which carries critical importance in the early diagnosis of skin cancer [3]. Dermoscopy is a non-invasive diagnostic method which allows for a closer examination of the pigmented skin lesion. It is performed with the help of an instrument called a dermatoscope. The procedure of dermoscopy allows for a visualization of the skin structure in the epidermis that would not otherwise be possible to the naked eye. Studies [127] suggest that a growing number of practitioners are incorporating dermoscopy into their daily practices. Dermoscopy can be categorized into three modes—polarized contact, polarized non-contact, and nonpolarized contact (unpolarized dermoscopy). Polarized and nonpolarized dermoscopy are complementary, and utilizing both to acquire clinical images increases the diagnostic accuracy [128]. These images can then be processed with the help of AI methods to assist in the diagnosis of skin cancer [132,133,134].

Even though the mortality rate of skin cancer is significantly high, early detection helps to bolster the survival rate to over 95% [5]. Deep learning models are generalizations of multi-layer perceptron models and are widely used due to their high accuracy in visual imaging tasks. There are two major promising paths for skin cancer detection in this research. The first is employing machine learning techniques and strategies to assist in the detection of skin lesions, and classifying them accordingly. The second, as this article discusses, is deep learning frameworks and model-based approaches being implemented in the recent advancements concerning skin cancer diagnosis. Table A1 in Appendix A contains a list of abbreviations used in this review, as well as their definitions.

### 1.1. Contribution of this Survey

We provide a comprehensive study of the various machine learning and deep learning models used for skin cancer diagnosis. Brief explanations of several machine learning and deep learning methodologies are included.

- This survey comprehensively discusses the application of various machine learning and deep learning methods in the implementation of skin cancer diagnosis.
- There is a discussion of new techniques in skin lesion detection such as deep belief networks and extreme learning machines, along with the traditional Computational Intelligence techniques such as random forests, recurrent neural networks, and k-nearest neighbors, etc.
- There is a designated tabular summary of works on the deep learning and machine learning techniques used for skin cancer diagnosis and detection. The tabulated summary also includes key contributions and limitations for the same.
- There is a classification of various types of skin cancer based on tumor characteristics that have been elucidated for a deeper understanding of the problem statement.
- The study also describes various open challenges present and future research directions for further improvements in the field of skin cancer diagnosis.

Table 1 presents a comparison between the current review and the previous review articles of machine-learning-based and deep-learning-based techniques in skin cancer diagnosis. The depth of the discussion in Table 1 has been used as a criterion for comparing different review articles. A high or H depth of the discussion indicates that the article contains a dedicated session for the said topic. A moderate or M depth of the discussion denotes that the review article has a subsection or a paragraph corresponding to the topic. A low or L depth of the discussion implies that the article has mentioned the topic, but not explained it comprehensively. A not discussed or N depth of the discussion indicates that the topic has not been covered in the article. 

### 1.2. Survey Methodology

#### 1.2.1. Search Strategy and Literature Sources

Repositories and databases such as IEEE, ScienceDirect, and PubMed, etc., were used to find relevant research studies and articles. The relevancy was determined based on the paper’s context (the central theme of the paper being the diagnosis of skin cancer based on AI/ML/DL models), the research paper’s title, abstract screening, keyword matching, and the conclusion of the study. The keywords employed were cancer diagnosis, skin cancer, deep learning, machine learning, skin lesion, melanoma cancer, and cancer detection, etc. A total of 1057 non-duplicate articles were found initially. Table 2 includes the search terms and the corresponding set of keywords associated with these terms.

#### 1.2.2. Inclusion Criteria

The articles included were primarily filtered based on their relevance. Apart from relevancy, only articles written in English were selected. Furthermore, only articles published after 2014 were considered for inclusion. 

#### 1.2.3. Elimination Criteria

The elimination of articles was based on abstract and introduction screening. Articles were then eliminated based on the quality of their research and the lack of references. The parameters used to judge the research quality were the reputation of the journal the article was published in, using metrics such as the h-index and impact factor, the date of publication (the older the date, the less relevant the article may be in present day), and the number of citations the research study had. In addition, any missing relevancy and the redundancy of the research were also considered in the elimination process.

#### 1.2.4. Results

**Figure 1:**
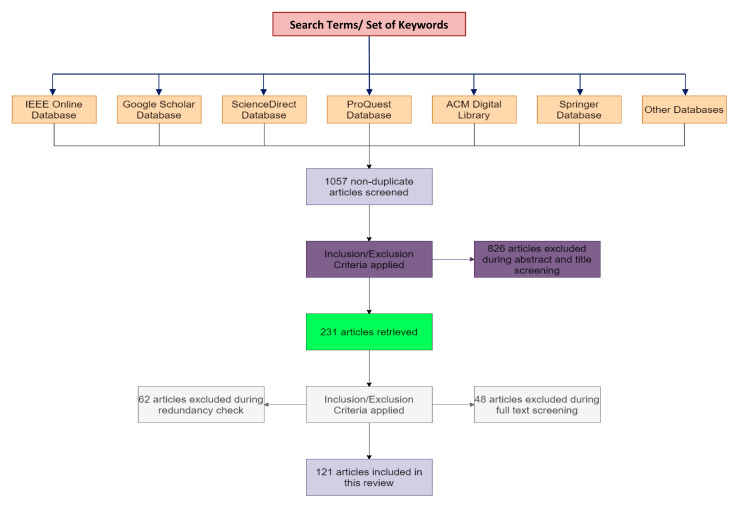
Flow diagram for the selection process of research articles using PRISMA method.

**Figure 2:**
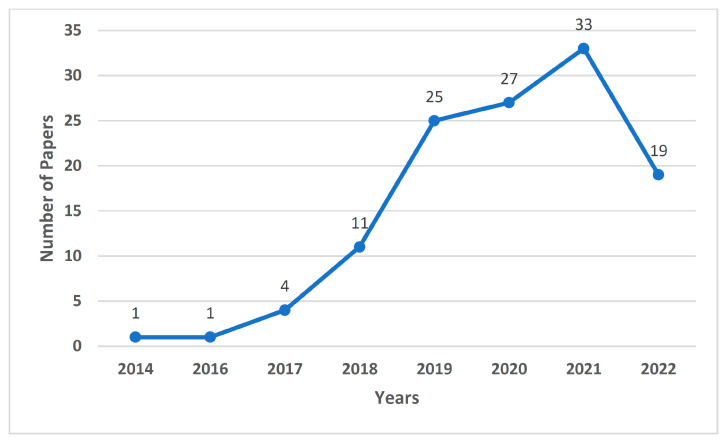
Number of papers per year, used in the review.

**Figure 3:**
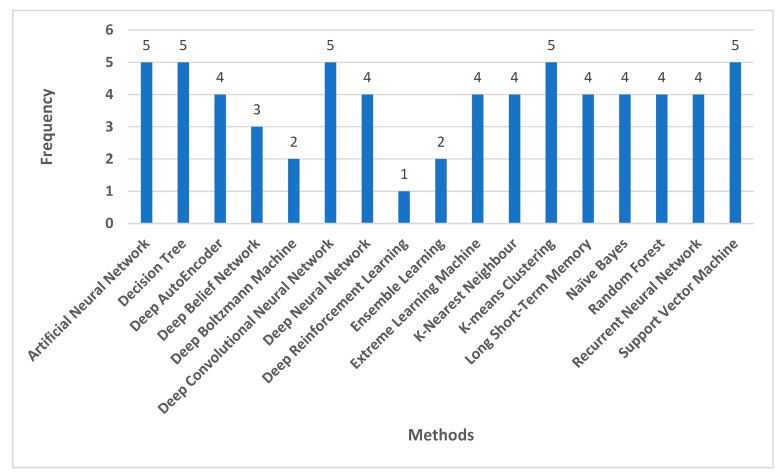
ML and DL methods versus frequency of papers used in this work.

Out of the 1057 non-duplicate articles filtered out from the various research repositories, 826 articles were excluded during the abstract and title screening. From the remaining 231 articles, 62 articles were excluded during the redundancy check and 48 articles were excluded during the full text screening. Finally, 121 articles were obtained after applying the inclusion/exclusion criteria. Figure 1 shows the PRISMA method implementation for the same. Figure 2 indicates the number of reference papers published in each year. Figure 3 demonstrates the various methods that this study encapsulates, and the number of papers cited corresponding to each methodology.

### 1.3. Structure of this Review

**Figure 4:**
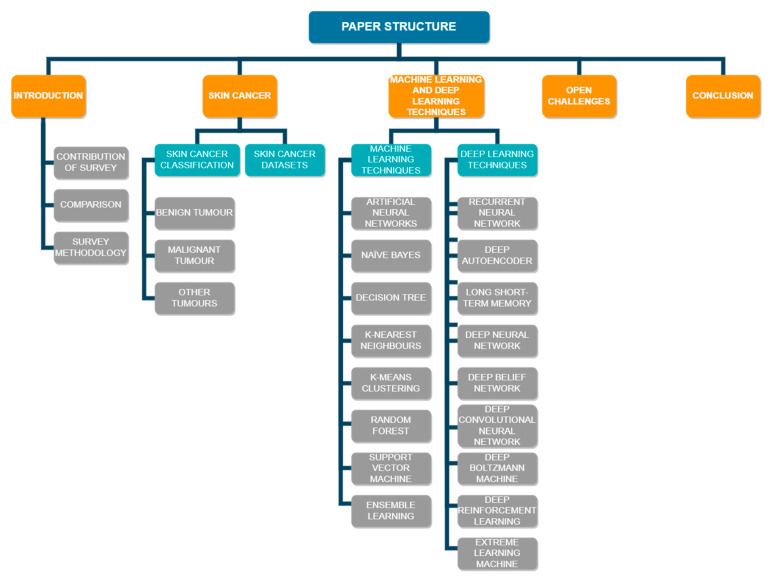
Structure of this review.

This paper is organized as follows. A comparison with previous reviews on skin cancer diagnosis and survey methodology for the same is covered in Section 1. Section 2 provides an overview of skin cancer, as well as the datasets commonly used in various studies in the field of skin cancer diagnosis. Section 3 is divided into two major subsections. The subsections describe the techniques used to diagnose skin cancer using machine learning and deep learning frameworks and algorithms, respectively. Section 4 talks about the open challenges faced in the field of skin cancer diagnosis, while Section 5 gives an insight into future research directions. The conclusion is given in Section 6, followed by the references used for this research. Figure 4 visualizes the structure of this study.

## 2. Skin Cancer

Skin cancer is associated with the abnormal growth of skin cells that are found either in the epidermis or the dermis. The skin cells are usually those that are exposed to sunlight, but skin cancer can also occur in those cells that are not ordinarily exposed [129]. This research focuses on the skin cancer that occurs in the epidermal cells, namely keratinocytes and melanocytes. Skin cancer can be largely divided into three subcategories.

1. Basal cell carcinoma: this type of cancer affects and originates from the basal cells. Basal cell carcinoma comes from keratinocytes, which are found in the epidermis. These may invade the entire epidermal thickness.
2. Squamous cell carcinoma: this subdivision deals with the uncontrollable growth of the abnormal squamous cells present in the root. Squamous cells are flat cells that are found in the tissue that constitutes the surface of the skin, and the lining of vital organs such as the respiratory organs, digestive tracts, and hollow organs of the body.
3. Melanoma: this form of cancer develops when melanocytes start to grow abnormally. Melanocytes are the cells that can become melanoma. Melanoma can develop anywhere in the skin, while it can also form in other parts of the body such as the eyes, mouth, and genitals, etc.

**Figure 5:**
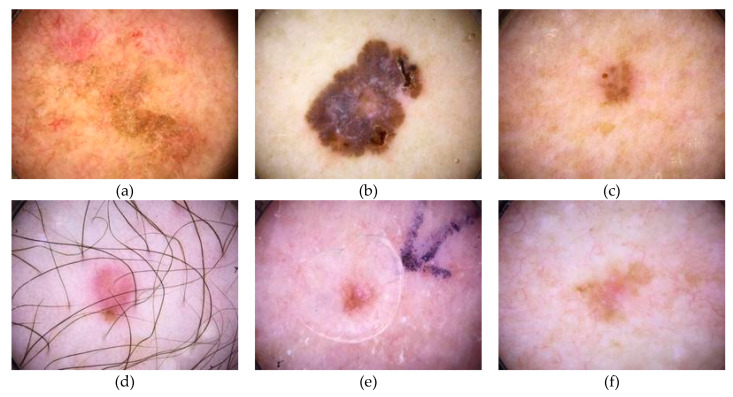
Dermoscopic sample images of skin cancer: (**a**) squamous cell carcinoma, (**b**) basal cell carcinoma, (**c**) benign dermatofibroma, (**d**) benign seborrheic keratosis, (**e**) benign actinic keratosis, and (**f**) malignant melanoma.

**Figure 6:**
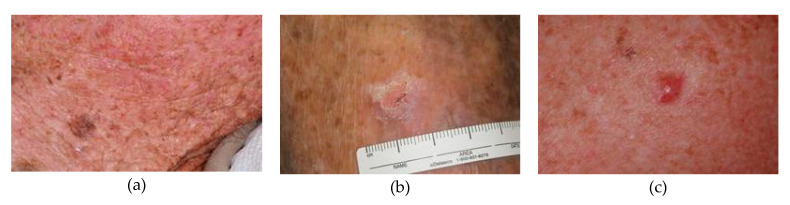
Clinical sample images of skin cancer: (**a**) malignant melanoma, (**b**) squamous cell carcinoma, and (**c**) basal cell carcinoma.

Figure 5 and Figure 6 include images from the International Skin Imaging Collaboration (ISIC) dataset to demonstrate the different types of skin cancer images that are available for training and testing. Figure 5 shows dermoscopic images, while Figure 6 displays clinical images from the skin cancer dataset.

Branching out of skin cancer are skin tumors, which are chiefly responsible for the mortality rate once diagnosed with the same [17]. Skin tumors can be categorized into two types, namely melanoma and non-melanoma. Irrespective of the technological advancements made in the field of curing cancer, to date, the early detection and diagnosis of any tumor combined with enough therapy leads the way to a successful treatment [18]. There are multiple ways to classify and categorize skin cancer. Most of them employ the use of deep learning techniques such as convolution neural networks in [19], while the others use specialized tools such as non-invasive imaging tools [20].

### 2.1. Skin Cancer Classification

When cells become cancerous, they start to grow uncontrollably due to various reasons, one such reason being a damaged cell DNA. This random behavior of the cell may lead to uneven accumulation and form a solid mass or lump, called a tumor. Tumors are often associated with uncontrollable growth in solid tissues such as muscles and bones. Tumors are further subdivided into two major categories, as described in the following section.

#### 2.1.1. Benign Tumor

Benign tumors are a collection of these cells that grow abnormally but are non-cancerous. According to [21], these tumors are generally classified according to their level of origin within the skin. The skin has three levels of subclassification in this regard, the epidermal layer, the dermal layer, and the subcutaneous layer. Another common taxonomy followed for benign tumors is based on the cell of their origin. Certain well-known examples are the melanocyte or the keratinocyte [22]. 

#### 2.1.2. Malignant Tumor

Malignant tumors are tumors that are cancerous. The affected cells metastasize through the bloodstream and the lymph nodes. In the context of skin cancer, malignant tumors emerging from the surface epithelium of the skin and the epidermis include cutaneous melanoma and non-melanoma cancers such as basal cell carcinoma [23]. Cutaneous melanomas constitute only 4% of all skin cancers, but they are by far the most significant ones, due to their lethality [24]. Ref. [25] conducted a study on developing deep learning techniques to help classify tumors as benign or malignant. False positives and negatives lead to a substandard prognosis of skin cancer. Article [26] discusses the challenges of detecting malignant tumors, which include, but are not limited to, noisy images, irregular tumor boundaries, and uneven image sizes. Hence, the need for deep learning and machine learning methods to detect malignant tumors is paramount.

#### 2.1.3. Other Tumors

The last subclassification of tumors is loosely classified as pre-malignant tumors. These cells are not cancerous at that moment of time, but they have the potential to become malignant. The major problem faced by the authors of [27] while detecting pre-malignant tumors was the scarcity of images. This led to them use the same dataset for training and validation. This does not come a surprise, as the study conducted in [28] also faced difficulty in recording pre-malignant lesion data. Pre-malignant tumors are often clubbed with certain malignant subtypes such as actinic keratosis, which is a squamous cell carcinoma despite being premalignant as well [25]. This makes it difficult to distinguish between the different classes of tumors.

### 2.2. Skin Cancer Datasets

Table 3 describes the various datasets used in previous studies and analyzes the constituents of each dataset. Furthermore, the table also identifies the skin cancer image categories available in the respective datasets.

## 3. Machine Learning and Deep Learning Models for Skin Cancer Diagnosis

### 3.1. Need for Machine Learning and Deep Learning Models for Skin Cancer Diagnosis

Artificial Intelligence has laid the foundation for integrating computers into the medical field seamlessly [30]. It provides an added dimension to diagnosis, prognosis, and therapy [36]. Recent studies have indicated that machine learning and deep learning models for skin cancer screening have been on the rise. This is primarily because these models, as well as other variants of Artificial Intelligence, use a concoction of algorithms, and when provided with data, accomplish tasks. In the current scenario, the tasks include, but are not limited to, the diagnosis of the patient, the prognosis of the patient, or predicting the status governing the ongoing treatment [37]. Diagnosis is the process of understanding the prevailing state of the patient, while prognosis refers to the process of predicting the future condition of the patient by extrapolating all the current parameters and their corresponding outputs. AI has now progressed to the point where it can be successfully used to detect cancer earlier than the traditional methods [6]. As early detection is key for a fruitful treatment and better outcome of skin cancer, the need for machine learning and deep learning models in the field of skin cancer is paramount.

### 3.2. Machine Learning Techniques

#### 3.2.1. Artificial Neural Networks

Artificial neural networks (ANNs) are systems that draw inspiration from the animal brain. ANNs have been used to predict non-melanoma skin cancer by inputting a certain set of tried and tested parameters fit for training, such as gender, vigorous exercise habits, hypertension, asthma, age, and heart disease etc. [38] The ANN takes the entire dataset as the input. To improve the accuracy of the model, the network inputs are normalized to values between 0 and 1. The outputs are treated as typical classification outputs, which return fractional values between 0 and 1. ANNs can also be used to detect skin cancer by taking an image input and subjecting it through hidden layers [39]. This process is carried out in four sequential steps, the first of which is to initialize random weights in the ANN system. Next, each of the activation values are calculated. Consequently, the magnitude of the error is also known as the loss change. The weights are updated proportionately, with respect to the loss. Until the loss reaches a certain lower bound or a floor value, the three steps are repeated. In this field that pertains to skin cancer detection, visual inspection is the introductory stage. This is due to the similarities shared between various subcategories of tumors, such as color, area, and distribution. Owing to this reason, the use of ANNs is encouraged to enhance multi-class skin lesion detection [40]. The trained network models are used with a logistic regression model to successfully detect skin lesions while reducing the false positives and negatives in the process. The choice of activation function for the ANN is completely dependent on the user, and it is to be noted that each function carries its own sets of advantages and disadvantages with respect to the convergence of the model and the computational load [40]. ANNs have been used to simultaneously predict various symptoms that generally occur in cancer-affected patients, as seen in [41]. The risk of symptoms predicted were that of pain, depression, and poor well-being. The input to the ANN was a list of 39 distinct covariates. The input features can be classified into five subcategories, such as demographic characteristics such as age and sex, clinical characteristics such as the cancer type and stage, treatment characteristics such as the radiation treatment and cancer surgery, baseline patient reported measures such as the performance status and symptom burden status, and finally, health care utilization measures such as whether the patient has been hospitalized or if they have a live-in caregiver. ANNs play an important role in predicting skin cancer and the presence of a tumor, due to their flexible structure and data-driven nature, owing to which they are considered as a potential modeling approach [42].

The model proposed by [38] reports a sensitivity of 88.5% and a specificity of 62.2% on the training set, while the validation set showed a comparable sensitivity of 86.2% and a specificity of 62.7%. Similarly, the ANN model in [39] was tested over multiple sets, each using an increasing number of training and testing image ratios. The accuracy returned by the model falls between 80% and 88.88%. 

In [38,39,40], emphasis is put on the need for optimizing predictors, increased model parameters, and the conduction of more clinical testing to improve the sensitivity and specificity of the model. Despite being easy to implement and cost effective, ANN models require further development in future studies for skin cancer diagnosis.

#### 3.2.2. Naïve Bayes

Naïve Bayes classifiers are probabilistic classifiers that work by employing the use of Bayes’ theorem. Naïve Bayes classifiers have been used in the field of skin cancer to classify clinical and dermatological images with high precision [43]. The model has reached an accuracy of 70.15%, as it makes use of important pieces of data to develop a strong judgement and assists physicians in the diagnosis and precise detection of the disease. Naïve Bayes classifiers extend their applications by providing a means to detect and segment skin diseases [44]. For each output class of the classifier, a posterior probability distribution is obtained. This process is performed iteratively, which implies that the method requires lesser computational resources, as it avoids the need for multiple training sessions. The Bayesian approach has also been used to probabilistically predict the nature of a data point to a high degree of accuracy, as seen in [45]. The final classification made in this case combines the existing knowledge of data points to use in the Bayesian analysis. The Bayesian sequential framework has also been put into use to aid models that help to detect a melanoma invasion into human skin. A total of three model parameters were estimated with the help of the model, namely, the melanoma cell proliferation rate, the melanoma cell diffusivity, and ultimately, a constant that determines the degradation rate of melanoma cells in the skin tissue. The algorithm learns data through the following, in a sequential manner: a spatially uniform cell assay, a 2D circular barrier assay, and finally, a 3D invasion assay. This Bayesian framework can be extracted and used in other biological contexts due to its versatile nature. This is chiefly possible in situations where detailed quantitative biological measurements, such as skin lesion extraction from scientific images, is not easy; hence, the extraction method must incorporate simple measurements from the images provided, like the Bayesian framework does [46]. 

Naïve Bayes classifiers, as discussed in [43], achieve an accuracy of 70.15% and a specificity of 73.33%. At the same time, the classifiers do not breach the 70% mark in sensitivity and precision. The accuracy appears to follow a similar pattern in naïve Bayes classifiers from other studies such as [44], where the diagnostic accuracies reported are 72.7%. 

The recurring scope of improvement in [43,44,45] revolves around experimenting with different color models, as well as using different types of dermal cancer datasets in the training. In [44,46], they elucidate the pressing need for further pre-processing before training naïve Bayes classifiers for skin cancer diagnosis.

#### 3.2.3. Decision Tree

Decision trees are a supervised learning method which are primarily used for classification problems and are occasionally extended to fit regression problem statements as well. Decision trees have been used to provide an intuitive algorithm that helps quantify the long-term risk of non-melanoma skin cancer after a liver transplant. This is done by utilizing the variables closely associated with the peri-transplant period [47]. The classifier is used as a view for the patients which provides personalized solutions such as chemoprophylaxis. A slight variation of decision trees can also be employed, as seen in [48]. The article proposes a random decision tree algorithm to detect breast surgery infection. The risk factors that came along with the algorithm in this case were obesity, diabetes, and kidney failure, etc. While the study investigates breast cancer, skin cancer is most closely associated with breast cancer due to the presence of the dangerous melanoma type. Decision trees showcase its versatility in the way it is used. In [49], decision trees are used as a mode for the visual representation of problem by dividing each branch into the different outcomes possible during a clinical procedure. The decision tree model was used to gauge the cost effectiveness of the sentinel lymph node biopsy, a new standard technique used in the treatment of melanoma and breast cancer. The cost effectiveness was measured with respect to head and neck cutaneous squamous cell carcinoma, a subsection of skin cancer. The decision tree presented outputs to determine whether the treatment was cost effective for a particular set of tumors, or if it could be used generally. Decision trees can also be used as an intermediate layer instead of keeping them as a standalone classifier. In [50], they demonstrate the effectiveness of this architecture in extracting regions and classifying skin cancer, using deep convolution neural networks. Most of the features are classified using decision trees and other counterpart algorithms such as support vector machines and k-nearest neighbors. Decision trees are also used to attain clarity in the classification of breast cancer, as can be seen from [51]. The error analysis of the proposed model reveals that the foundational decision tree models provide users with easy-to-use outcomes and a very high degree of clinical detection and diagnostic performance, as compared to its predecessors. 

The decision tree model from [47] reports a specificity of 42% and a sensitivity of 91%. Similarly, the models presented in [48] return a sensitivity, specificity, and accuracy greater than 90%. This trend follows suit in the model proposed by [50], where all the three parameters cross 94%. On the contrary, models like those in [49] return a slightly lower sensitivity of 77% but report a 100% specificity. 

Decision trees’ model predictions are heavily dependent on the quality of the datasets. The common pitfalls encountered by [47,50] are that the model testing and training datasets had an identical distribution of variables; hence, this eliminates the prospect of training the model on entirely independent cohorts.

#### 3.2.4. K-Nearest Neighbors

The k-nearest neighbors algorithm, also referred to as the KNN, is a parametric supervised classification algorithm that uses distance and proximity as metrics to classify the data points. KNNs were used as an evaluation algorithm to detect skin cancer and melanomas. The KNN model was then used to produce a confusion matrix which helps with visualizing the accuracy of the entire model [52]. Apart from this case of use, KNNs have also been used extensively by extending the model as per requirement. In [53], they extend KNN to use the Radius Nearest Neighbors classifier to classify breast cancer and calculate the evaluation metrics such as accuracy and specificity. The reason for augmenting the KNN solely lay in the limitations posed by an extreme value of k. For a small k, the KNN classifier is highly sensitive to outliers, and for a large value of k, the classifier underfits on the training data points. This problem is overcome by normalizing the radius value of each point to recognize outliers effectively. The applications of KNNs have been expanded by using them for detecting the anomalous growth of skin lesions [54]. KNNs are hybridized with Firefly to provide quantitative information about a skin lesion without having to perform any unnecessary skin biopsies. The hybrid classifier built upon KNN is used to predict and classify using two primary methods: threshold-based segmentation and ABCD feature extraction. The Firefly optimization coupled with KNN helps to recognize skin cancer much more effectively than its predecessors, while keeping computational and temporal complexity to a minimum. To classify and discriminate between melanoma and benign skin lesion in clinical images, ref. [55] made use of multiple classifiers, out of which the KNN classifier returned competent results. The article also makes use of different color spaces and tests the classifiers on each of them to demonstrate the feasibility of the algorithms to detect melanomas in various color spaces. 

The KNN classifiers of [52], with the number of neighbors set to 15, returned an accuracy of 66.8%, with a precision and recall for positive predictions of 71% and 46%, respectively. The recall value increases almost twofold for negative predictions, while the precision score for the same lingers around 65%. The values in [53] provide a different perspective to the modified KNN classifiers, as they report an accuracy of over 96%. Fuzzy KNN classifiers, as shown in [54], have an accuracy of 93.33%, with a sensitivity of 88.89% and a specificity of 100%.

Despite being a viable approach to diagnosing skin cancer, KNN classifiers require the provision to be trained continually, as suggested by [52]. Furthermore, with the dearth of feasible datasets, the size of suitable training data proves to be a limitation for [52,53]. To mitigate the adverse effects of minimal training data, the KNN classifier can fit into an online learning method that builds over time and keeps learning as and when the classifier acquires more data.

#### 3.2.5. K-Means Clustering

K-means clustering is a clustering method that is grouped under unsupervised learning. By employing a fuzzy logic with the existing k-means clustering algorithm, studies have been conducted on segmenting the skin melanoma at its earliest stage [56]. Fuzzy k-means clustering is applied to the pre-processed clinical images to delineate the affected regions. This aids the process to subsequently be used in melanoma disease recognition. K-means clustering has widespread cases of use and can be used to segment skin lesions, as seen in [57]. The algorithm groups objects, thereby ensuring that the variance within each group is at minimum. This enables the classifier to return high-feature segmented images. Each image pixel is assigned a randomly initialized class center. The centers are recalculated based on every data point added. The process is repeated until all the data points have been assigned clusters. Unlike a binary classifier like k-means, where each data point can belong to only one cluster, fuzzy c-means clustering enables the data points to be a part of any number of clusters, with a likelihood attached to hit. The fuzzy c-means algorithm outputs comparatively better results in comparison with the legacy k-means clustering algorithm. Fuzzy c-means provide a probability for data points that depends on the distance between the cluster center and the point itself. In [58], fuzzy c-means were used in place of the k-means algorithm to detect skin cancer, inspired by a differential evolution artificial neural network. The simulated results indicated that the proposed method outperformed traditional approaches in this regard. The k-means algorithm can also be used as an intermediate layer to produce outputs, as trained on by deep learning methods. In [59], they demonstrated an algorithm where k-means were used to segment the input images based on the variation of intensities. The clusters thus formed were then subjected to further processing to aid in the detection of melanoma cancer. The traditional k-means algorithm can also be used to detect skin lesions. To augment the quality of the results, it can be used with a gray level co-event matrix, a local binary pattern, and red, green, and blue color modes [60]. K-means clustering is heavily dependent on external factors being extracted successfully, such as color features, lesion orientation, and image contrast [56,58,59]. This engenders the need for coherency in the diagnosis pipeline that utilizes the k-means clustering. The pipeline must accurately extract external features before the clustering algorithms take them as input.

K-means clustering models tend to return a high detection accuracy. For instance, the model that extends fuzzy logic, like in [56], returns an accuracy of over 95%. Other k-means clustering models, like those in [58] and [59], also report a detection accuracy of 90%.

#### 3.2.6. Random Forest

Random forests are an extension of decision trees. They are an ensemble learning method commonly used for classification problems. Random forests extend their applications to detect skin cancer and classify skin lesions, as done in [61]. Random forests permit the evaluation of sampling allocation. The steps followed in the proposed method are to initialize a training set. The training set is then bootstrapped to generate multiple sub-training sets. By calculating the Gini index for each of the sub-training sets, the model is then populated with decision values. The individual decision values are then combined to generate a model that classifies by voting on the test samples. Skin cancer can also be classified by characterizing the Mueller matrix elements using the random forest algorithm [62]. The random forest algorithm builds various sub-decision trees as the foundation for classification and categorization tasks. Every individual decision tree is provided with a unique logic that constitutes the binary question framework used in the entirety of the system. In comparison with the original decision tree, the random forest provides enhanced results while reducing the variance bias. This helps to prevent the overfitting of the data, which was otherwise seen in decision trees. Other studies in the classification of skin cancer involve classifying the dermoscopic images into seven sub-types. This has been implemented with the help of random forests [63]. The procedure to create a random forest is slightly unconventional in this study. After preparing a dataset to train on, the random forest is then amassed by arranging a relapse tree. The ballot casting is conducted after the forest architecture is built. The different types of sub-classifications that the random forest was trained on were basal cell carcinoma, benign keratosis lesion, dermatofibroma, melanocytic nevi, melanoma, and vascular types. Similarly, skin lesion classification has also been performed with the help of random forests and decision trees in [64]. Using random forests are key since predecessor algorithms lack the reliability aspect of skin image segmentation and classification. The random forest is generated by selecting a subset of random samples in the skin lesion dataset. For each feature in the subsets, a decision tree is created to get a prediction. A voting process is then established for each of the prior outputs, and a forecast result with the most votes is selected as the final step. 

Random forest classifiers, as seen in [61], report an accuracy, sensitivity, and specificity of around 70%, regardless of the features incorporated to segment the required area, such as ABCD rule or GLCM features. Depending on the dataset used, random forest classifiers can also have a high accuracy while detecting skin cancer. The models in [62] achieved an average accuracy of 93%.

The features of a random forest classification algorithm are invariant to image translation and rotation [61,64]. This allows future research to be more liberal with its datasets and extend them to a variety of geographies to discern the consistency in results returned by skin cancer classification.

#### 3.2.7. Support Vector Machine

Support vector machines (SVMs) are supervised learning models that help classify, predict, and extrapolate data by analyzing them. SVMs can be used to classify different types of skin lesions. In [65], ABCD features are used for extracting the characteristic features like shape, color, and size from the clinical images provided. After selecting the features, the skin lesion is classified with the help of SVMs into melanoma, seborrheic keratosis, and lupus erythematosus. This method of using ABCD along with SVM generates great results while delivering significant information. For a narrower classification, SVMs have also been used to classify skin lesions as melanoma or non-melanoma [66]. The process was divided into six phases: acquiring the image, pre-processing the image, segmentation, extracting the features, classifying the image, and viewing the result. From the experiment, the features extracted were texture, color, and shape. To extend the nature of the above model, SVMs have also been employed to identify and detect carcinoma or infection in the early stages before it aggravates [67]. The chief difference in the extension and itself lies in the feature extraction procedure. In [67], they pre-process the input image by employing grey scale conversion and then chaining the resultant image with noise removal and binarization subprocesses. The region of interest is removed in segmentation to help with accurate classification. Similarly, for the early detection and diagnosis of skin cancer, a bag-of-features method was used, which included spatial information. The SVM was developed with the help of a histogram of an oriented gradient optimized set. This resulted in encouraging results when compared to state-of-the-art algorithms [68]. By using Bendlet Transform (BT) as features of the SVM classifier, unwanted features such as hair and noise are discarded. These are removed using the preliminary step of median filtering. BT outperforms representation systems such as wavelets, curvelets, and contourlets, as it can classify singularities in images much more precisely [69].

The average accuracy of the SVM classifier models presented in [65] was about 98%, while the sensitivity and specificity averaged to 95%. The SVM model in [66] also had all three parameters greater than 90%. 

#### 3.2.8. Ensemble Learning

Ensemble learning is a machine learning model that combines the predictions of two or more models. The constituent models are also called ensemble members. These models can be trained on the same dataset or can be suited to something completely different. The ensemble members are grouped together to output a prediction for the problem statement. Ensemble classifiers have been used for diagnosing melanoma as malignant or benign [70]. The ensemble members for the same are trained individually on balanced subspaces, thereby reducing the redundant predictors. The remaining classifiers are grouped using a neural network fuser. The presented ensemble classifier model returns statistically better results than other individual dedicated classifier models. Furthermore, ensemble learning has also been used in the multi-class classification of skin lesions to assist clinicians in early detection [71]. The ensemble model made use of five deep neural network models: ResNeXt, SeResNeXt, ResNet, Xception, and DenseNet. Collectively, the ensemble model performed better than all of them individually.

#### 3.2.9. Summary of Machine Learning Techniques

Analyzing the various implementations of machine learning models in the field of skin cancer diagnosis indicates that simple vector machines are undoubtedly the most precise and accurate models. The main caveat of using SVMs is the need for the meticulous pre-processing of input data. In terms of user flexibility, k-means clustering and k-nearest neighbors lead the way, without compromising much on accuracy and performance. KNNs, however, require to be trained continuously as more data points are added. This might prove to be quite tedious as the volume of input data is highly irregular and cannot be predicted. Naïve Bayes models have the lowest accuracy of all the machine learning techniques studied in this paper, and understandably so, as various techniques make use of the fundamentals of the naïve Bayes theorem and develop it further, such as decision trees and random forests. Decision trees perform decently but are highly dependent on the quality of the dataset, which is an uncontrollable variable in the system. Random forests do not have the provision to learn image rotation and translation on the fly, reflecting the same in their classification accuracy. Depending on the dataset used, random forests can either perform really well, or get only around 50% of classifications correct. ANNs, being the steppingstone for various techniques to develop, suggest that while the results may be good, they cannot be increased further. ANNs have reached a saturation point in terms of the modifications made, and other techniques must be employed if any improvement is expected. Ensemble models, although complicated and tough to implement, return accuracies higher than the models taken individually for the multi-class classification. 

**Figure 7:**
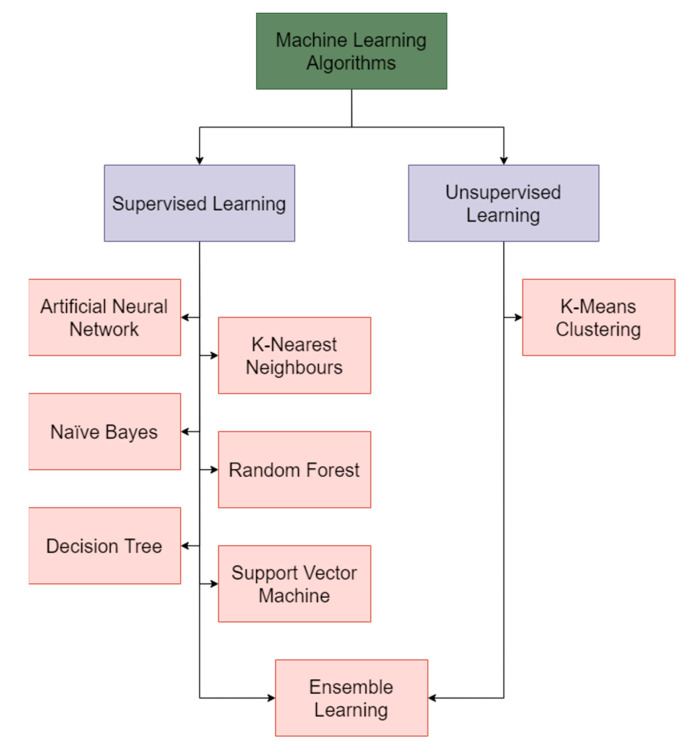
Current machine learning models in skin cancer diagnosis: tree illustration.

Table 4 provides an executive summary of the machine learning techniques used in the diagnosis of skin cancer. Figure 7 conceptualizes the machine learning models in skin cancer diagnosis discussed in this study. 

### 3.3. Deep Learning Techniques

#### 3.3.1. Recurrent Neural Network

A recurrent neural network (RNN) is categorized as a subdivision of artificial neural networks. RNNs have been used in the detection of melanoma skin cancer [72]. The classification phase of the proposed model employs deep learning techniques by combining the optimization notion into an RNN. The existing region growing algorithm and RNN have been improved by using them alongside the modified deer hunting optimization algorithm (DHOA). Apart from standalone models, RNNs have also been used in ensemble models alongside convolution neural networks, as seen in [73], to classify skin diseases. Predecessor models were unable to use the long-term dependence connection between key image features and image classes. This served as the motivation for the proposed model. Deep features are extracted from the clinical images, after which the features are fed into the dual bidirectional long short-term memory network to learn the features. Ultimately, a SoftMax activation function is used to classify the images. Similarly, ensemble models can also be used to automate the detection of mammogram breast cancer [74]. Just like in [73], the first step involves feature extraction through the grey level co-occurrence matrix and the grey level run-length matrix. These two are then given to the RNN layer as inputs, and the tumor segmented binary image is provided as input to the convolution neural network layer. The two independent classifiers’ results show an improved diagnostic accuracy. RNNs have also been used in the segmentation of various dermoscopic images [75]. The reason for incorporating a recurrent model is primarily due to its ability to train deeper and bigger models. Furthermore, recurrent models ensure better feature representation and ultimately, better performance for the same number of parameters.

Modified RNNs, as proposed in [72], have an average accuracy of slightly over 90%, with an F1-score of 0.865. By varying the variable value in the equation used, the accuracy follows a linear trend by increasing as the value increases. Like the previous result, the RNNs in [74] have an accuracy of 98% but an F1-score of 0.745. The model in [75] reports a testing accuracy of 87.09% and an average F1-score of 0.86.

#### 3.3.2. Deep Autoencoder

Deep autoencoders are neural networks that are trained to emulate the input as the output. They consist of two symmetrical deep belief networks. In the field of skin cancer, deep autoencoders have been used for reconstructing the dataset, which is then used to detect melanocytes by employing spiked neural networks [76]. The structure of the autoencoder model consists of three main layers: the input layer, hidden layers, and the output layer. The model is run on the foundational principle that every feature is not independent of each other, otherwise it would compromise the efficiency of the model. Autoencoders have also been used to recognize and detect melanoma skin disease [77]. The various autoencoders used were Deeplabv3+, Inception-ResNet-v2-unet, mobilenetv2_unet, Resnet50_unet, and vgg19_unet. Quantitative evaluation metrics showed that the Deeplabv3+ was a significant upgrade from the other architectures used in the study to detect melanoma skin. Skin cancer detection has also been carried out with the help of custom algorithms involving autoencoders, such as the social bat optimization algorithm [78]. The detection process takes place in three steps. Firstly, the clinical images are pre-processed to remove the noise and artefacts present. The pre-processed images are then fed to the feature extraction stage through a convolution neural network and a local pixel pattern-based texture feature. Right after this stage, the classification is completed using a deep stacked autoencoder, much like the evaluation in [77,79] of different autoencoders for skin lesion detection. The five architectures evaluated in this study are u-net, resu-net, vgg16unet, desnenet121, and efficientnetb0. Among the evaluated architectures, the densenet121 architecture showed the highest accuracy.

The autoencoder-based dataset used in [76] returned an average accuracy of 87.32%, with the sensitivity and specificity within one point of accuracy as well. The study in [77] concluded that using autoencoders consistently increased the accuracy and F1-score in various datasets, as opposed to the models that did not employ deep autoencoders. The average accuracy of the models in [77] after using autoencoders is around 94%. In a similar fashion, the deep stacked autoencoder presented in [78] returned an average accuracy of 93%, sensitivity of 84%, and specificity of 96%. 

#### 3.3.3. Long Short-Term Memory

Long short-term memory, or LSTM, is an artificial neural network that uses feedback connections to enable the processing of not only single data points, but also sequential data. LSTM has helped in classifying skin diseases by efficiently maintaining stateful information for accurate predictions [80]. The robustness of the proposed algorithm helps to recognize target regions faster, while using almost half the number of computations compared to predecessor algorithms. The use of LSTM further bolsters the accuracy of prediction due to its previous timestamp retention properties. Other than plain recognition, LSTMs can also be used to predict cancer and tumors in irregular medical data [81]. This is made possible by the enhanced overall performance of LSTMs in screening time series data. The risk groups being dealt with in the proposed study correlated well to the temporal cancer data (time to cancer diagnosis). Skin disease classification models have been designed using deep learning approaches like LSTM with the assistance of hybrid optimization algorithms such as the Hybrid Squirrel Butterfly Search Optimization algorithm (HSBSO) [82]. The modified LSTM is developed by implementing the HSBSO and the optimized parameters of an LSTM model to maximize the classification accuracy. LSTMs help in improving the overall efficiency of the proposed skin disease classification model. Deep learning models are not only limited to the clinical images of tumors. Certain studies demonstrate the usage of convolutional LSTMs to detect aneurysms on angiography images [83]. The angiography images are obtained from the 2D digital subtraction angiography, thereby making it hard to distinguish cerebral aneurysms from the overlapping vessels. The convolutional LSTM (C-LSTM) is a variant of the LSTM. Each LSTM cell has a convolutional operation associated with it. C-LSTM inherits the advantages of LSTM while being very suitable for the analysis of spatiotemporal data due to its internal convolution architecture. In real-life diagnoses, physicians combine lateral and frontal sequences to aid the decision-making process. Employing a similar concept, the C-LSTM is fed with two inputs: frontal and lateral images to increase the spatial information, consequently improving the performance of the entire system.

The incorporation of LSTM components to pretrained models such as the MobileNet V2, as seen in [81], outperforms some state-of-the-art models, with a training accuracy of 93.89% and validation accuracy of 90.72%. The study conducted in [82] demonstrated that LSTM performs better than most machine learning models, with an average sensitivity of 53% and specificity of 80%. 

#### 3.3.4. Deep Neural Network 

Deep neural networks are those neural networks that expand to a certain level of complexity and depth. Vaguely, the certain level is decided to be two or more layers. Deep nets have been used to estimate the uncertainty lying in skin cancer detection [84]. The motivation behind the model lies in the ineptness of publicly available skin cancer detection software for providing confident estimates of the predictions. The study proposes the Deep Uncertainty Estimation for Skin Cancer (DUNEScan) that provides an in-depth and intuitive analysis of the uncertainty involved in each prediction. Deep nets have also been used to classify skin cancer at a dermatological level [85]. The classification of skin lesions, with the help of images alone, is an arduous task due to the minute variations in the visual appearance of lesions. Deep nets show immense potential for varied tasks that constitute multiple fine subcategories. The performance of the model is evaluated using biopsy-proven clinical images that were classified into two binary classification problems: keratinocyte carcinomas and benign seborrheic keratoses, and malignant melanomas and benign nevi. The deep net model achieves a performance that matches and, in some cases, outperforms all the experts associated with the evaluation program. For instance, the confusion matrix comparison between deep nets and dermatologists (experts) exhibits similarities in the misclassification of tumors [85]. The distribution demonstrates the difficulty in classifying malignant dermal tumors for both experts as well as deep nets, but also shows that experts noticeably confuse benign and malignant melanocytic lesions with each other, while the deep net classifies it with a higher degree of accuracy. Deep nets are usually implemented as a single-stream network. Two-stream deep nets, on the other hand, combine two recognition streams to handle the separate features associated with the input data. Two-stream deep nets have been used to design intelligent systems that classify skin cancer [86]. The two streams in the proposed method are a fusion-based contrast enhancement technique coupled with a pretrained DenseNet201 architecture, and down sample the extracted features using the proposed selection framework. The evaluation parameters suggest that the proposed method returns an improved performance upgrade over the predecessor models. Deep net models have also been deployed in real world applications, empowering medical professionals by assisting the process of diagnosing skin cancer and employing a prediction model for over 100 skin disorders [87]. The deep learning algorithms have proven to be a successful method with which to diagnose malignant tumors, as well as suggest treatment if trained with a dataset consisting of substantial numbers of Asian and Caucasian populations. Using a convolution neural network as the ancillary tool, the performance is elevated and can be used to diagnose cutaneous skin diseases.

#### 3.3.5. Deep Belief Network

Deep belief networks (DBN) are generative graphical models that are composed of multiple layers of latent variables. DBNs have been used for cancer prediction, as can be seen in [88]. They perform the model training in two steps. Firstly, each layer is separately trained in an unsupervised manner. This is done to retain the maximum feature information. Subsequently, the output features are taken and used to train the entity relationship classifier in a supervised manner. DBNs have been designed to automatically detect regions of breast mass and diagnose them as benign, malignant, or neither [89]. The proposed DBN performs comparatively better than its conventional counterparts. This is because the conventional approaches depend on the output of selection feature algorithms. On the contrary, all the features were directly used without any reduction in their dimensions for the DBN model. To improve the diagnosis of skin melanoma by using DBNs in place of the traditional approach, dermoscopy has been studied [90]. The deep belief learning network architecture disperses the weights and hyperparameters to every position in the clinical image. By doing so, this makes it possible to scale the algorithm to varying sizes. The images are first use a Gaussian filter to remove the high and low intensities from the images. Subsequently, the pre-processed images are segmented using the k-means algorithm. The resultant images are then classified as per the output format of the proposed DBN.

The DBN presented in [88] reports a diagnostic accuracy of 81.48%. According to the study, for the evaluation criteria tested, the DBN outperformed the RNNs and CNNs, which had an accuracy of 73% and 68%, respectively. DBNs that are used to complement computer-aided diagnosis, as seen in [89], report an average accuracy of around 91%. For unsegmented images, the DBN model in [90] achieves an accuracy of 73% while the same model, when subjected to segmented images, achieves an accuracy of 90%. This suggests that DBNs might accurately predict if the input is segmented and pre-processed correctly.

#### 3.3.6. Deep Convolutional Neural Network

Convolutional neural networks (CNNs) are artificial neural networks that are primarily used in image processing and recognition. Deep convolutional neural networks have been implemented to classify skin cancer into four different categories: basal cell carcinoma, squamous cell carcinoma, actinic keratosis, and melanoma [91]. The methodology involves two methods, an error-correcting output codes simple vector machine (ECOC SVM) classifier, and a deep CNN. The authors use accuracy, sensitivity, and specificity as evaluation parameters. A slight variation from the previous method introduces a LeNet-5 architecture along with a deep CNN to classify the image data [92]. The model aids the diagnosis of melanoma cancer. The experiment results indicate that training data and number of epochs for training are integral to the process of the detection and diagnosis of melanoma cancer. Results suggest that training the model for over 100 epochs may lead to overfitting while training it for below 100 epochs leads to underfitting. In addition, there are several parameters which account for the accuracy of the results, such as the learning rate, number of layers, and dimensions of the input image. Since dermatologists use patient data along with deep CNNs for an increased diagnostic accuracy, recent studies have investigated the influence of integrating image feature data into the deep CNN model [93]. The commonly used patient data were sex, age, and lesion location. To accommodate the patient data, one-hot encoding was performed. The key differentiator between fusing the image features was the complexity associated with each classification, respectively. The studies indicate the potential benefits and advantages of amalgamating patient data into a deep CNN algorithm. Region-based CNNs have been employed to detect keratinocytic skin cancer on the face [94]. The algorithm aims to automatically locate the affected and suspected areas by returning a probabilistic value of a malignant lesion. The deep CNN was trained on over one million image crops to help locate and diagnose cancer. While the algorithm demonstrated great potential, certain pitfalls were highlighted: skin markings were mistaken as lesions by the deep CNN model. Secondly, the testing data usually made use of the physician’s evaluation data, rather than the clinical photographs alone, which ultimately led to the need for a multimodal approach. The developments of recent studies have enabled newly designed models to outperform expert dermatologists and contemporary deep learning methods in the field of multi-class skin cancer classification, using deep CNNs [95]. The model was fine-tuned over seven classes in the HAM10000 dataset. While ensemble models increase the accuracy for classification problems, they do not have a major role in refining the performance of the finely-tuned hyperparameter setup for deep CNNs.

The deep CNNs, as seen in [91], could classify skin cancer with an accuracy of 94.2%. Furthermore, the sensitivity and specificity of the model were also above 90%. Region-based CNN that is used to classify skin cancer on the face [94] returns an average accuracy of 91.5%. The study further emphasized the benefits of using a CNN-based model as a screening tool to improve public health, as the sensitivity of the general public was merely 50%. The model, on the other hand, averaged a sensitivity of 85%. 

#### 3.3.7. Deep Boltzmann Machine

Deep Boltzmann machines (DBM) are probabilistic, unsupervised, and generative models that possess undirected connections between multiple layers within the model. Multi-modal DBMs have been proposed to monitor and diagnose cancer before the mortality rate rises [96]. The multi-modal DBM learns the correlation between an instance’s genetic structure. The testing and evaluation phase use the same to predict the genes that are cancer-causing mutations specific to the specimen. By combining restricted Boltzmann machines (RBM) and a skin lesion classification model through optimal segmentation, the OS-RBM model helps to detect and classify the presence of skin lesions in clinical images [97]. The OS-RBM model carries out certain steps sequentially: image acquisition, pre-processing using Gaussian filters, segmenting the pre-processed images, extracting the features, and classifying the images. Segmenting images is executed through the Artificial Bee Colony algorithm.

#### 3.3.8. Deep Reinforcement Learning

Reinforcement learning (RL) is a training method often associated with rewarding and punishing the desired and undesired behaviors, respectively. Reinforcement learning algorithms have been incorporated into the medical scene to automatically detect skin lesions [98]. This is done by initially proceeding from coarse segmentation to sharp and fine results. The model is trained on the popular ISIC 2017 dataset and HAM10000 dataset. The regions are initially delineated. By tuning the hyperparameters appropriately, the segmentation accuracy is also boosted. As deep RL methods have the capability to detect and segment small irregular shapes, the potential for deep RLs in the medical background is immense.

#### 3.3.9. Extreme Learning Machine

Extreme learning machines (ELM) are essentially feedforward neural networks. While they provide a good generalization performance, the major difference arises in the learning speed. ELM models have been proposed to tackle the existing problem of skin cancer detection [99]. This detection takes place by differentiating between benign and malignant lesions. Upon pre-processing the clinical images, the regions are segmented using the Otsu method. The model optimizes and learns with the help of a deep belief network which introduces a Thermal Exchange Optimization algorithm. Using hybrid pretrained models along with ELMs for diagnosing skin cancer has also been researched [100]. The proposed diagnostic model makes use of the SqueezeNet model for the batch normalization layers. The layers towards the end of the model are replaced by ELMs. The ELMs are usually linked with a metaheuristic, for instance, the Bald Eagle Search Optimization metaheuristic, that enable the model to converge much faster than its contemporary counterparts. Instead of pretrained models, hybrid deep learning models have also been combined with extreme learning machines to classify skin lesions into multiple classes [101]. While majority of the steps remain the same, the major differences lie in the deep feature extraction that uses transfer learning and feature selection, which makes use of hybrid whale optimization and entropy-mutual information algorithms. Extreme learning machines can also be modified and used as an extreme gradient boosting method for the remote diagnosis of skin cancer [102]. Apart from diagnosis, the model also helps in the process of health triage. The major problem faced by the authors were the unbalanced categories in the dataset. To overcome this imbalance, data augmentation was incorporated. Integrating the skin lesions with the clinical data reinforced the accuracy and efficacy of the model.

ELM models that are used for multi-class skin lesion classification [101] produce high-quality predictions with an accuracy of over 94%. ELM models have been shown to consistently outperform respective benchmark studies, as seen in [102]. Even though the accuracy of the model in [102] hovers around 77%, it is significantly higher than the benchmark studies for the same set of data and conditions. When coupled with data augmentation, ELMs can avoid the risk of overfitting. 

#### 3.3.10. Summary of Deep Learning Models

Deep learning models provide robust solutions for skin cancer detection. Recurrent neural networks can accurately predict the incidence of skin cancer to a fairly high degree, but they come with the limitation of being efficient only when using large datasets. For smaller data points, RNNs will not have enough data to learn the features and predict as accurately. Autoencoders serve as a recourse for insufficient data. Deep autoencoder-based datasets, used with pretrained models, return highly accurate results. The major drawback involved in deep autoencoders is the parameter value initialization. Most of the studies employ a preliminary trial method to settle for the initial parameter values, which may prove to be infeasible for large models. Long short-term memory models outperform other deep learning techniques in terms of classification and tumor growth progress analysis, but the accuracy of the model sharply drops to below 80% when the quality of the images is substandard, such as poor illumination or conditions different from those in the testing dataset. Deep neural networks produce good results but cannot match the versatility of other deep learning techniques such as RNNs or LSTMs. DNN models find it tough to distinguish between blurry shadows or irregular borders unless they have been trained on such data. To be widely adopted, DNNs require training images with adequate quality, making it a cause of concern, as clinical data may not emulate the training dataset conditions. Deep belief network models return highly accurate results, but in similar conditions, are often outperformed by convolutional neural networks. CNNs provide users with the flexibility to extend the model with different learning techniques, as well as accurately predict different types of skin cancer. Most of the studies involving CNNs reported an average accuracy of over 90%. New techniques in the deep learning space make use of extreme learning machines. These models outperform state-of-the-art techniques, with reported accuracies of over 93%. While they return accurate results, they are susceptible to poorly augmented datasets, which can sharply decrease the accuracy of the model. 

**Figure 8:**
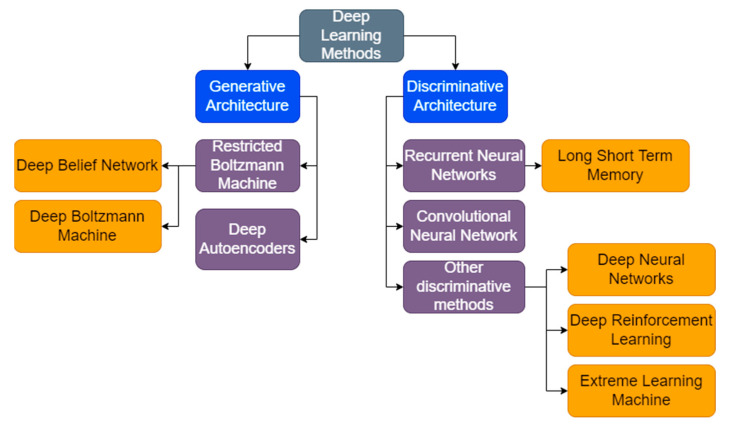
Current deep learning models for skin cancer diagnosis: tree illustration.

Table 5 summarizes the works discussed on deep learning models used for skin cancer diagnosis. Figure 8 shows the deep learning models in skin cancer diagnosis, as elucidated in this study. 

## 4. Open Challenges in Skin Cancer Diagnosis

### 4.1. Communication Barrier between AI and Dermatologists

Giant strides in the field of Artificial Intelligence for skin cancer diagnosis mean it may have established its place in the scene for years to come, but it has still not been able to breach the communication barrier that exists between dermatologists and AI. In [103], it is suggested that dermatologists must embrace the potential shown by AI applications in various fields, such as clinical and research situations. The preconceived notion surrounding the use of AI in the cancer diagnosis domain that the introduction of technology may eventually downsize the workforce, has set the wrong precedent, and has brought about apprehensions about adopting AI for the same. It must be understood that, while AI has been ever improving and returns a higher accuracy with respect to diagnoses, clinicians are undoubtedly more skillful in identifying mimetics, as well as patterns that have not been made available to the models through the training datasets [103]. The authors of [103] reiterate that the role of dermatologists is not limited to detecting and identifying skin lesions, but also to extract valuable information and inferences from their observations. At present, the latter is not quite fine-tuned and is still in the rudimentary stages in machines. In a survey conducted on Chinese dermatologists [104], the majority of the participants believed that AI in the workplace assisted with daily activities involving diagnosis and treatment. In accordance with the claims made by the authors of [103], the survey results indicated that only 3% of the dermatologist participants believed that AI could and would replace their day-to-day work. Another survey in [105] indicated positive diagnosis results and a higher accuracy, as compared to traditional approaches, after dermatologists used the help of AI in making their decisions. The need to bridge the communication gap between AI and dermatologists is paramount, and efforts must be taken to involve the various functionalities offered by AIs in the workplace. 

### 4.2. Dataset Availability and Features

Incorporating deep learning into cancer diagnosis in the real world comes with one major roadblock—the lack of availability of datasets. Machine learning and deep learning algorithms require huge amounts of datasets to be trained upon, without which the models will ultimately return subpar results. Some databases do not include benign lesions such as blisters and warts; these lesions are managed by dermatologists daily, making it a very common occurrence in day-to-day diagnoses. This poses the threat of missing skin cancer among benign lesions, as they are not included in the datasets. According to [7], most of the online publicly available datasets consist of only raw images. This essentially means that most of the headway must be generated by the researchers themselves. To tackle the prevalent issue of an imbalance in the datasets, researchers have started employing data augmentation techniques such as cropping, rotation, and filtering, which, in turn, increases the number of training images the models can use [8]. While the datasets provide a rich source of information for the researchers, the absence of clarity in the metadata for various characteristics, such as ethnicity and skin types, inhibits the utility of clinical images [33]. The future of datasets and the furthering of improvement in AI-based diagnostic methods have already been set in motion in the form of open science, such as providing clinical decision support for diagnoses and screening. To overcome the problems faced in obtaining datasets to a greater extent, the adoption of open science must gain traction.

### 4.3. Patient Perspectives on Artificial Intelligence

Artificial Intelligence is assured to change the way patients interact with healthcare-associated processes, but it has remained elusive in terms of patients’ outlook and perspectives on AI in healthcare. The survey conducted in [106] aims to decipher the reception of Artificial Intelligence in healthcare by the patients. The central theme of most responses revolved around the diagnosis of the illness. This establishes a symbiotic relation between patients and the use of AI. While around 75% of the patients were keen to recommend AI in healthcare to family members, the apprehension of the remaining 25% is concerning. One way to tackle this is by ensuring that the response to conflict can be resolved by seeking a biopsy to complement the Artificial Intelligence decision-making. Apart from incorrect diagnoses, patients also are wary about their medical and clinical history being made available if used for datasets. Without properly labeled images, AI will be unable to train properly, leading to incorrect diagnosis results. Data analytics involving AI models for skin cancer diagnoses heavily use these labels to infer observations for future research [35]. By concealing the data, or submitting falsified information, this destroys the purpose of training an AI. This can be mitigated by ensuring that data collection organizations maintain non-disclosure policies.

### 4.4. Variation in Lesion Images

Skin cancer diagnosis using machine learning and deep learning involves multiple steps, out of which the primary step is skin lesion segmentation. As straightforward as it may seem, this task is commonly associated with setbacks that inhibit its smooth completion, namely the variations in lesion sizes, imprecise and indistinct boundaries, and different skin colors. The variation in different images, such as illumination difference, leads to uneven shadows and bright areas, making it tougher to segment the skin lesions [107]. Conventional algorithms such as CNNs and CNN-based approaches may perform superiorly in terms of labeling, but they still return a poor contrast between lesion and regular skin images. This is due to the deviations in the dataset, such as skin tone and aberrations, etc. The immense variation in datasets leads to the lack of inference drawn from the results, as suggested in [108]. A varying methodological quality shows higher amounts of specificity and sensitivity when compared to an expert’s diagnosis, thus rendering the use of AI in healthcare less useful. The corresponding diagnostic score and criteria for qualifying as an expert have been covered in a review that studies optical coherence while diagnosing adult skin cancer [108]. To tackle the problem of the varying characteristics of skin lesions, AI models must look to maximize their diversification and intensification [109]. By employing such mechanisms, the models can overcome the stagnation faced due to the increasing variation. In addition, the diagnosis of skin cancer in people of color is put off until advanced stages, due to the variations in lesion images that engender from the difference in skin tones [122]. Furthermore, due to socio-economic factors such as care barriers, models are not trained on different skin tones [122]. They develop an inherent bias towards the dominant skin tone and lesion color that the model has been trained on, which ultimately compromises the quality of the skin cancer diagnosis. 

### 4.5. Dermatological Image Acquisition

Image acquisition in dermatology generally deals with close-up images of lesions or dermatosis. In most cases, the anatomical context of such images is lost due to the exclusion of surrounding structures, while the primary focus of the image is the lesion. Furthermore, with the rapid adoption of digital skin imaging applications, the utilization of smartphone-acquired images in dermatology have also increased proportionally [123]. While many studies have proposed methods to detect melanomas from inconsistent dermoscopy images, most of them produce localized results that cannot be used universally due to the acquisitive conditions they are trained on, such as isolated datasets and specific illumination conditions, etc. [124]. In addition to these problems, the quality of the acquired images is significantly compromised due to the varying illumination conditions during the acquisition phase, such as specular reflection. In [125], a generative adversarial neural network is proposed to deal with the persisting present problem of color inconsistency. The wider adoption of such methods, and an increase in the novelty of ideas that overcome erratic dermoscopy images, are required for overcoming the challenges faced by image acquisition.

### 4.6. Ethical and Legal Perspectives

While the use of AI in the clinical practice and healthcare domains has lots of upsides, it raises many ethical challenges. The use of health AI, albeit for transforming the patient–clinician relationship, carries ambiguity around the use of informed consent. The clinicians are unaware about the circumstances under which they should or should not inform the patient about AI being a part of the relationship [110]. AI decisions carry lots of weight, and they come with the challenges of safety and transparency. While it is understood that no AI model can be correct all the time, incorrect decisions can prove to be fatal, as well as mold correct decisions as unsafe. This gives rise to the concern around model algorithms’ fairness and bias. Models are trained on a particular set of data, making them biased to the characters they inferred from the dataset. It is virtually impossible for any dataset to exactly sample the world’s population and thus raises a flag for a cause for concern. AI technology has been identified to have a tremendous capability to threaten patient preference [111]. Parallelly, the use of health-related AI inevitably intersects with the law in more than one way. The question of how liability should be attributed in the case of harmful treatment is still unanswered. Similarly, AI bias against historically disadvantaged groups can attract anti-discrimination and human rights laws [112]. It is yet unknown whether the existing privacy laws can protect patients undergoing AI-based healthcare. It is necessary to understand that, while the potential benefits of AI in healthcare are plenty, it cannot be adopted for commercial use unless the ethical and legal challenges are responded to, as they serve as the bedrock of the entire system.

**Figure 9:**
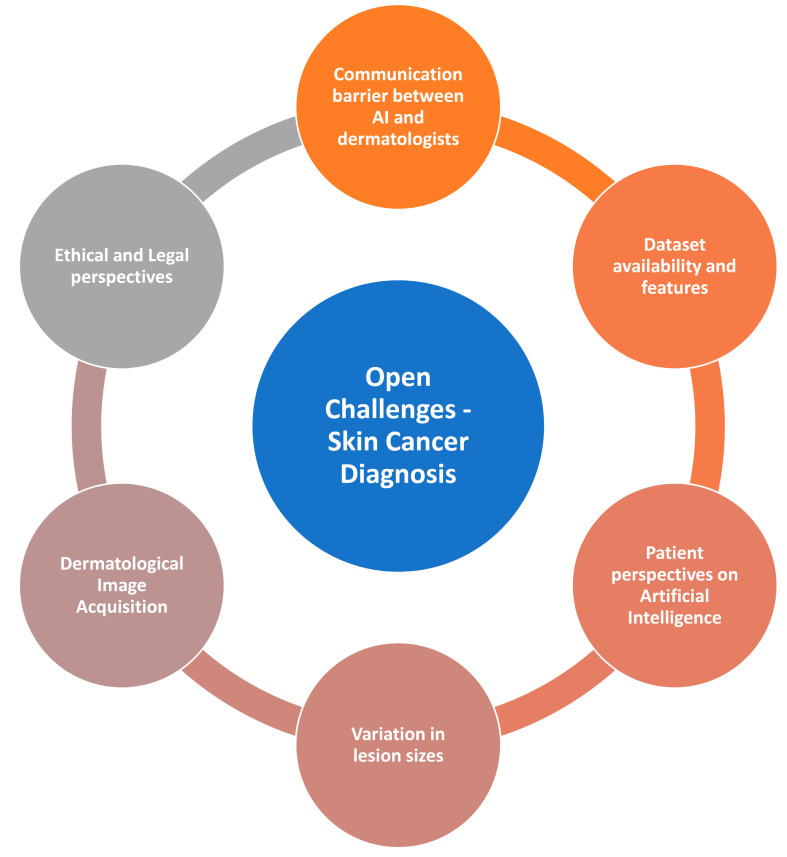
Open challenges in skin cancer diagnosis.

Figure 9 visualizes the open problems in skin cancer diagnosis using machine-learning- and deep-learning-based techniques. 

## 5. Future Research Directions

### 5.1. Combining AI with Next-Generation Sequencing for Refining Skin Cancer Diagnosis

Next-generation sequencing (NGS) technologies have progressed to facilitate the increase in data output and the efficiencies associated with it. NGS is categorized based on its respective read lengths [113]. NGS is used to determine the order of nucleotides in entire genomes or targeted regions in RNA and DNA. High-throughput DNA sequencing technology and methods have paved the way for commercializing new techniques [114]. The goal of DNA sequencing methods is to support speed while staying accurate, coupled with lower input rates of DNA and RNA input data. Squamous cell carcinoma (SCC) carries one of the highest tumor mutation burdens amongst all cancers. By employing the targeted next-generation sequencing of localized and metastatic high-risk SCCs, gene mutations can be compared with the intention of identifying key differences and improving targeted treatment alternatives [115]. Since the introduction of molecular tools, the discovery of new viruses, such as the papillomavirus, has been accelerated. NGS can be combined with improved protocols to help detect known and unknown human papillomaviruses [116].

### 5.2. AI-powered Automated Decision Support Systems for Skin Cancer Diagnosis

Decision support systems are computerized programs used to assist with decision-making and choosing the right course of action; Artificial-Intelligence-powered decision support systems can be used in the diagnosis of skin cancer. They provide options of flexibility in designing deep learning classification models by hinting at the common procedures and looping patterns [117]. Support systems can be initialized with pretrained deep neural network models combined with transfer learning to classify skin lesion localization and classification [118]. Present day decision support systems are fused with automated deep learning methods. These methods are fine-tuned and trained with the help of transfer learning using imbalanced data [119]. The model extracts the features using an average pooling layer, although the extracted features are not sufficient. By employing a modified genetic algorithm based on metaheuristics, relevant and significant features are extracted which can further be sent to a classifier that acts as a decision support system. As mentioned in [120], using AI-powered decision support systems can help clinicians diagnose and potentially replace invasive diagnostic techniques.

### 5.3. Smart Robotics for Skin Cancer Diagnosis

Robots can be used to improve the detection of skin cancer. Existing robots like Vectra WB360 combine 3D images with the corresponding sequential digital dermoscopic images, owing to the non-invasive tracking of melanoma and non-melanoma skin cancer. The photographic analysis of the WB360 allows for a global view of the skin surface and generates a body map to record the evolution of the lesions. This feature is very useful in detecting any suspicious developments promptly.

### 5.4. Wearable Computing for Skin Cancer Diagnosis

Wearable computing is a paradigm that involves the computation of accessories that can be worn by humans. Any small device capable of computing and processing data that can be worn on the body is categorized as a wearable computer. Wearable computers have been used in the field of cancer detection [121]. A few challenges that have not allowed for widespread clinical adoption yet are the high fragility, bendability, non-cooperative form factor of the sensors used, inappropriate connectivity, clinical inertia, and ultimately the awareness, as well as the cost, associated with wearable devices. Future research and corporations should aim to reduce the cost policy while tackling the challenges of using wearable computing as a vital alternative for cancer detection.

These event-driven tools are beneficial when concerning computational effectiveness, higher efficiency, power consumption, flexibility, and improved real-time performance. The possibility of incorporating these tools into wearable devices could be beneficial in terms of performance enhancement.

### 5.5. Teledermatology

Teledermatology is described as technology that supports healthcare from a distance [130]. Teledermatology aims to provide clinical services to patients, monitor their health, and provide resources through remote locations. This technology can be used for diagnosing, screening, and even managing skin cancer effectively. In total, three prospective solutions for implementing these are the store and forward method, real-time video conferencing, and a hybrid solution that includes both. At present, the store and forward method is widely in use with patients taking pictures and videos, which are then forwarded to the dermatologist. The ease of the convenience and the inexpensiveness make it a very popular choice. Real-time video conferencing uses the interaction between patient and physician through a video calling software to provide immediate advice. This simulates an in-person clinic experience, where the physician can verify medical data and history before prescribing anything. This method requires a stable internet connection and a high-quality video camera if it must be used for skin cancer. A low-quality camera may not fully capture the border and may eventually lead to erroneous diagnoses. The hybrid method mixes the advantages of both the methods: real-time video conferencing coupled with high-quality images sent to the dermatologist. Together, these serve as a beneficial way to consult and diagnose skin cancer. Teledermatology can be used with many machine learning and deep learning techniques to make the entire process much smoother. For instance, there are various CNN architectures which can be employed using transfer learning [131]. Doing so can help the dermatologist make decisions with a higher degree of confidence. Ensemble models can be incorporated into the pipeline for the store and forward method, enabling the system to be more accurate after the patient sends adequate-quality photos or videos. 

**Figure 10:**
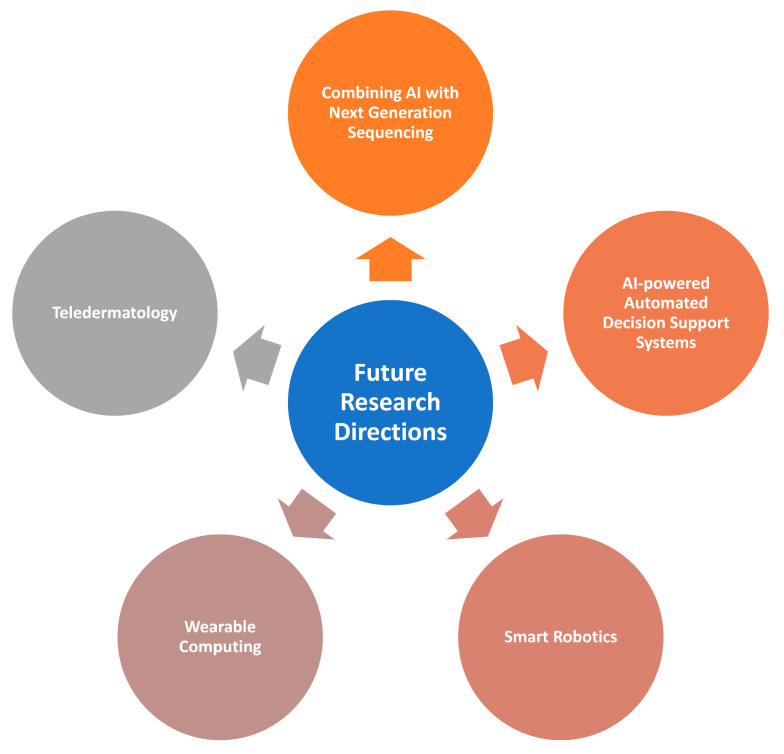
Future research directions of skin cancer diagnosis.

Figure 10 illustrates the future directions for machine-learning- and deep-learning-enabled skin cancer diagnosis.

The internet of medical things (IoMT) and cloud computing will be the essential elements in upcoming mobile AI-powered healthcare-related decision support systems. In this framework, the event-driven tools can be beneficial when concerning computational effectiveness, real-time compression, data transmission efficiency, power consumption, and flexibility [135,136,137]. The possibility of incorporating these tools into contemporary mobile healthcare solutions can be investigated in the future.

## 6. Conclusions

A comprehensive survey is presented on machine learning and deep learning techniques, deployed for an automated skin cancer diagnosis. A comparison is made to the widely used skin cancer datasets and dominant studies. An insight discussion is had while exploring the lessons from prior works. Its aim is to set this survey as a benchmark for further studies in the field of skin cancer diagnosis by also including the limitations and benefits of the previous works. It is concluded that the Artificial Intelligence (AI)-based healthcare solutions come with many pre-requisites, dependencies and issues that must first be resolved before they can scale up. The AI research carries ethical and legal ambiguities, along with a lack of clinical data on all skin types, thereby inducing unintended bias in the model’s prediction. Moreover, although AI is gaining traction in the dermatology discipline, it still has lots of room to grow and enhance further in terms of the sensitivity, specificity, and accuracy of detecting the skin lesions. Additionally, dermatologists must take the first step in accepting and embracing AI, not as a threat to their professions, but as an ancillary tool to complement their diagnoses. While considering the challenges for implanting end-to-end AI-based solutions in healthcare, there are lots of prospects, promises, and challenges. Wearable computing and robotics are evolving, and AI healthcare can be incorporated into these recent innovations to ease the apprehensive market. While the limited available data suggest a parity between those who are keen on adopting AI healthcare, and those aversive towards it, the room to improve automated skin cancer detection has been established.

## Figures and Tables

**Table 1:** Comparison of the current review with the previous reviews in AI-powered skin cancer diagnosis.

Reference	Year	One-Phrase Summary	Machine Learning Models in Skin Cancer Diagnosis	Deep Learning in Skin Cancer Diagnosis	Open Challenges in Skin Cancer Diagnosis	Future Directions for Skin Cancer Diagnosis
Our review	-	A comprehensive survey on machine learning and deep learning techniques used to diagnose skin cancer	H	H	H	H
[11]	2022	A review on cancer diagnosis using Artificial Intelligence	H	H	M	N
[12]	2022	A research article on the recent advancements in cancer diagnosis using machine learning and deep learning techniques	H	H	L	M
[6]	2021	A review of machine learning and its applications in the field of skin cancer	H	L	M	H
[7]	2021	A minireview on deep learning and its use in cancer diagnosis and prognosis prediction	N	H	M	H
[10]	2021	A review on skin disease diagnosis with deep learning	N	H	N	H
[14]	2021	A review on skin cancer classification via convolution neural networks	N	M	M	N
[15]	2021	A survey on deep learning techniques for skin lesion analysis and melanoma cancer detection	N	H	M	N
[9]	2020	A review article on Artificial-Intelligence-based methods for diagnosis of skin cancer	M	M	H	N
[13]	2020	A review on malignant melanoma classification using deep learning	N	H	M	H
[16]	2020	A survey in cancer detection using machine learning	H	N	H	H
[8]	2019	A bibliographic review on cancer diagnosis using deep learning	N	H	M	N

Depth of discussion: L—low, M—moderate, H—high, N—not discussed.

**Table 2:** Search terms.

Search Term	Set of Keywords
Skin	skin cancer, skin disease, skin cancer diagnosis, skin cancer detection, skin lesion
Cancer	cancer type, cancer diagnosis
Deep	deep learning, deep neural networks
Melanoma	melanoma skin cancer, melanoma cancer
Machine	machine learning
Machine learning techniques	artificial neural network, naïve Bayes, decision tree, k-nearest neighbors, k-means clustering, random forest, support vector machines, ensemble learning
Deep learning techniques	recurrent neural networks, deep autoencoders, long short-term memory, deep neural network, deep belief network, deep convolutional neural network, deep Boltzmann machine, deep reinforcement learning, extreme learning machine

**Table 3:** List of various skin cancer datasets employed by previous studies.

Reference	Creator and Year of Dataset	Skin Cancer Categories	Dataset Used	Dataset Size	Type of Data	Details About the Dataset
[132]	International Skin Imaging Collaboration, 2020	Actinic keratosis, basal cell carcinoma, dermatofibroma, melanoma, nevus, seborrheic keratosis, squamous cell carcinoma, vascular lesion	ISIC	2357 images	Dermoscopic images	Contains images of malignant and benign oncological diseases. Melanoma and mole images are slightly dominant in the dataset
[133]	Nilsel Ilter, H. Altay Guvenir, 1998	Melanoma and non-melanoma	DermIS, DermQuest	72 images in DermIS and 274 images in DermQuest	Not reported	Contains lesion images. They are subject to various artifacts such as drastic shadow effect and differing illumination.
[134]	Tschandl, P., 2018	Actinic keratoses and intraepithelial carcinoma, basal cell carcinoma, benign keratosis-like lesions, dermatofibroma, melanoma, melanocytic nevi, and vascular lesions	HAM10000	10015 images	Dermoscopic images	More than half of lesion images are validated through histopathology. Remaining images are confirmed through expert consensus or in-vivo confocal microscopy.
[35]	Dongtan Sacred Heart Hospital, Hallym University, and Sanggye Paik Hospital, Inje University, 2016	Basal cell carcinoma	Hallym	152 images	Dermoscopic images	Country of origin is South Korea and a total of 106 members participated in the creation of this dataset
[35]	Department of Dermatology at Asan Medical Center, 2017	Basal cell carcinoma, squamous cell carcinoma, intraepithelial carcinoma, and melanoma	Asan Dataset	17125 images and 1276 test images	Clinical images	While the thumbnails were available for free downloading, the full-size images required external permission and it came at a cost of US $200 or £145.
[34]	Mitko Veta et al., 2016	Not reported	TUPAC 2016 Dataset	500 training and 321 test images	Whole slide images	Images to predict tumor proliferation scores from whole slide images.

**Table 4:** Summary of works on machine learning techniques in skin cancer diagnosis.

Reference	Skin Cancer Category	Machine Learning Model	Description of Approach Used	Dataset	Key Contribution	Limitations	Performance Evaluation Metrics and Results
[38]	Non-melanoma skin cancer	Artificial neural network	12 neurons in each layer, inputs normalized to fall between 0 and 1, sigmoid activation function	National Health Interview Survey Dataset (NHIS 2016)	Multi-parametrized artificial neural network	Model does not include ultraviolet radiation exposure and family history data while making predictions	AUC is area under ROC curve. Training AUC—0.8058, validation AUC—0.8099
[44]	Skin disease detection and segmentation	Naïve Bayes classifier	Skin lesion segmentation using a dynamic graph cut algorithm followed by a naïve bayes classifier for skin disease classification	ISIC 2017	Flexible group minimizing for alike functions, making them decipherable in polynomial time	Cannot differentiate between certain colors	Diagnostic accuracy–72.7%, sensitivity–91.7%, specificity-70.1%
[47]	Non-melanoma skin cancer	Decision tree	Cox regression analysis to identify variables that enter the decision tree analysis	Oregon Procurement Transplant Network STAR 2016	Confirms importance of known risk factors and also identifies new variables establishing risk of getting non melanoma skin cancer	Model building and validation sets were not from independent cohorts	Cumulative incidence rate highest risk group: 7.4%, intermediate risk group: 3.1–5.5%, lowest risk group: 0.8%
[54]	Skin lesion	K-nearest neighbor classifier	Firefly with k-nearest neighbor algorithm to predict and classify skin cancer using threshold-based segmentation	-	Recognize skin cancer without performing unnecessary skin biopsies	Image pre-processing and segmentation is heavily dependent on threshold values	False predictive value: 0.0, false negative rate: 11.11%, sensitivity: 88.89%, specificity: 100%
[56]	Melanoma skin cancer	K-means clustering	Region-based convolutional neural networks along with fuzzy k-means clustering.	ISIC 2016, ISIC 2017, PH2	Fully automated skin lesion segmentation at its earliest stage	Model is heavily reliant on successful segmentation from the R-CNN stage	Sensitivity: 90%, specificity: 97.1%, accuracy: 95.4%
[61]	Melanoma skin Cancer	Random forest	Watershed segmentation used for feature extraction and then classified with random forest	ISIC	Section lesions on skin with increased precision	Same classification can be carried out with higher accuracy using a simple vector machine	Accuracy: 74.32%, sensitivity: 76.85%, specificity: 71.79%
[66]	Melanoma skin cancer	Simple vector machine	Extracted features such as texture, color, shape are inputs to the SVM classifier for skin lesion classification	University Medical Center Groningen (UMCG) database	Computer Aided Diagnosis support system for image acquisition, pre-processing, segmentation, extraction, classification, and result viewing	No support for hair removal and image cropping techniques, classification model can be improved further	Confusion matrix: [3,7,62,64], where [true positive, true negative, false positive, false negative] sensitivity: 90%, specificity: 96%
[71]	Multi-class skin cancer	Ensemble learning	Weighted average ensemble learning based model using 5 deep learning models	Human Against Machine (HAM10000), ISIC 2019	Significantly improved result as compared to models individually and existing systems	Trained over a highly imbalanced dataset leading to compromised results while testing and validation	Confusion matrix, ROC-AUC score

**Table 5:** Summary of works on deep learning models in skin cancer diagnosis.

Reference	Skin Cancer Category	Deep Learning Model	Description of Approach Used	Dataset	Key Contribution	Limitations	Performance Evaluation Metrics and Results
[72]	Melanoma skin cancer	Recurrent neural networks	Classification phases uses modified deep learning algorithm by coalescing optimization concepts from RNNs	PH2	Superior to existing algorithms in terms of optimal segmentation and classification for melanoma skin cancer	Heavy dependence on parameters for segmentation and classification	Algorithmic analysis including specificity: 0.94915, sensitivity: 0.83051, precision: 0.89091, F1-score: 0.85965, etc.
[76]	Skin cancer detection	Autoencoders	Dataset is reconstructed using autoencoder model, reconstruction and spiking networks contribute to enhanced performance	ISIC	Feature sets obtained from convolution model are suitable for merging	Model extracts many unnecessary and irrelevant features	Specificity: 0.9332, sensitivity: 0.9372, precision: 0.9450, F1-score: 0.9411, accuracy: 0.9354
[81]	Skin cancer diagnosis	Long short-term memory model	Tumor marker data values were used to train and test an LSTM model	Two independent medical centers	LSTM model demonstrates superiority while dealing with irregular data and can be used when time intervals between tests vary	Inability to analyze irregular tumor marker data for cancer screening	Time-to-cancer diagnosis in different risk groups, risk stratification
[87]	Binary classification, multi-class skin cancer diagnosis	Deep neural network	CNN architectures trained on large datasets and evaluated against algorithm-assisted clinicians’ results	Edinburgh and SNU datasets	Model serves as an ancillary tool to enhance diagnostic accuracy of clinicians	Outcome of algorithm is significantly affected by composition of input images; performance is sub-optimal if input image quality is low	Improvement in sensitivity and specificity by 12.1% and 1.1%, respectively
[88]	Malignant tumor detection	Deep belief network	Analyze patient data from deep learning perspective, merged with patient attributes and case reports to construct an expert system helping to predict the probability of early cancer	Jiangsu Provincial Hospital of Traditional Chinese Medicine	Relatively effective dimensional reduction and noise cancellation technique, reduces missed clinician diagnoses during endoscopy and treatment	Medium runtime in comparison to other deep learning methods	Accuracy: 0.8148, precision: 0.8571, recall: 0.6, F1 score: 0.7059
[91]	Melanoma, carcinoma, keratosis	Deep convolutional neural network	Classifies skin cancer using ECOC SVM and deep CNN, images are cropped to reduce noise	Pretrained on ImageNet, Internet Images for fine-tuning	Multi-class skin cancer classification using fine-tuned pretrained ImageNet model	Model does not extend to ABCD (asymmetry, border, color, diameter) rule	Accuracy: 0.942, specificity: 0.9074, sensitivity: 0.9783
[96]	Tumor causing somatic mutations	Deep Boltzmann machine	Multi-modal deep Boltzmann machine approach for prediction of somatic mutation genes that undergo malignant transformation, model learns relation between germline and mutation profiles using data	-	Genome-based diagnostic test to monitor for the presence of cancer-driving mutations	Sample size of is limited, Whole Exome Sequencing (WES) data displayed at gene level	Average accuracy: 0.7176, *p*-value
[99]	Melanoma skin cancer	Extreme learning machine	After pre-processing, Otsu method is employed to segment region of interest, subsequently, feature extraction is applied to mine important characteristics, deep belief network is used to categorize and classify	ISIC for training, SIIM-ISIC melanoma for validation	Optimized Pipeline feature designed for efficient detection of melanoma from images, DBN uses Thermal Exchange Optimization Algorithm as new meta-heuristic method	Computationally very intensive and time consuming	Accuracy: 0.9265, specificity: 0.8970, sensitivity: 0.9118, PPV: 0.8676, NPV: 0.9412

## Appendix A

**Table A1:** List of abbreviations used in this manuscript along with their full form.

Acronym	Definition
AI	Artificial Intelligence
ANN	Artificial neural network
KNN	K-nearest neighbors
ABCD	Asymmetry, border, color, diameter
SVM	Support vector machine
ROC	Receiver Operating Characteristic
AUC	Area under curve
RNN	Recurrent neural network
DHOA	Deer hunting optimization algorithm
LSTM	Long short-term memory
DBN	Deep belief network
CNN	Convolutional neural network
DBM	Deep Boltzmann machine
RL	Reinforcement learning
ELM	Extreme learning machine
NGS	Next generation sequencing
DNA	Deoxyribonucleic acid
RNA	Ribonucleic acid
SCC	Squamous cell carcinoma
